# One-Pot Method for Preparation of Magnetic Multi-Core Nanocarriers for Drug Delivery

**DOI:** 10.3390/ma12030540

**Published:** 2019-02-12

**Authors:** Črt Dragar, Tanja Potrč, Sebastjan Nemec, Robert Roškar, Stane Pajk, Petra Kocbek, Slavko Kralj

**Affiliations:** 1 Department for Materials Synthesis, Jožef Stefan Institute, 1000 Ljubljana, Slovenia; crt.dragar@gmail.com (Č.D.); sebastjan.nemec@ijs.si (S.N.); 2 Faculty of Pharmacy, University of Ljubljana, 1000 Ljubljana, Slovenia; Tanja.Potrc@ffa.uni-lj.si (T.P.); robert.roskar@ffa.uni-lj.si (R.R.); Stane.Pajk@ffa.uni-lj.si (S.P.); Petra.Kocbek@ffa.uni-lj.si (P.K.); 3 Laboratory of Biophysics, Jožef Stefan Institute, 1000 Ljubljana, Slovenia

**Keywords:** magnetic nanocrystals, magnetic drug delivery, nanocarriers, multi-core particles, magnetic nanoparticles, drug release

## Abstract

The development of various magnetically-responsive nanostructures is of great importance in biomedicine. The controlled assembly of many small superparamagnetic nanocrystals into large multi-core clusters is needed for effective magnetic drug delivery. Here, we present a novel one-pot method for the preparation of multi-core clusters for drug delivery (i.e., magnetic nanocarriers). The method is based on hot homogenization of a hydrophobic phase containing a nonpolar surfactant into an aqueous phase, using ultrasonication. The solvent-free hydrophobic phase that contained tetradecan-1-ol, γ-Fe_2_O_3_ nanocrystals, orlistat, and surfactant was dispersed into a warm aqueous surfactant solution, with the formation of small droplets. Then, a pre-cooled aqueous phase was added for rapid cooling and the formation of solid magnetic nanocarriers. Two different nonpolar surfactants, polyethylene glycol dodecyl ether (B4) and our own *N*^1^,*N*^1^-dimethyl-*N*^2^-(tricosan-12-yl)ethane-1,2-diamine (SP11), were investigated for the preparation of MC-B4 and MC-SP11 magnetic nanocarriers, respectively. The nanocarriers formed were of spherical shape, with mean hydrodynamic sizes <160 nm, good colloidal stability, and high drug loading (7.65 wt.%). The MC-B4 nanocarriers showed prolonged drug release, while no drug release was seen for the MC-SP11 nanocarriers over the same time frame. Thus, the selection of a nonpolar surfactant for preparation of magnetic nanocarriers is crucial to enable drug release from nanocarrier.

## 1. Introduction

The continuous development of novel approaches to materials synthesis offers innovative solutions for many of the present challenges in the life sciences [1,2,3]. Nanocrystals are the building blocks of composite or hybrid nanostructures, and they offer an innovative option to solve important challenges in medical diagnostics and therapeutics [4,5,6]. Hybrid nanostructures are composed of different nanocrystals and other functional components (e.g., polymers, lipids) to form individual nanocarriers. Among a number of promising inorganic nanocrystals, only magnetic iron oxide nanocrystals have generally been recognized as safe for human use by the regulatory agencies [7]. However, they should be precisely designed to avoid reactive oxygen species (ROS) generation in vivo. It was recently shown that the production of ROS was greatly dependent on a number of factors, including the size of the nanoparticles, concentration, surface properties and, importantly, the coating used [8,9].

Iron oxide nanocrystals are used commercially as a negative contrast agent for nuclear magnetic resonance (NMR) imaging and in the treatment of hyperthermia. When iron oxide nanocrystals are exposed to an alternating magnetic field, they can produce and release heat to targeted cancerous tissue, such as glioblastomas [10,11,12,13,14,15]. Although intensive research has been focused on the development of magnetically-responsive drug-delivery systems over the last few decades, no such system has arrived on the nanomedicine market to date.

The design and preparation of efficient magnetically-responsive drug-delivery systems are faced with many challenges. Frequently, active pharmaceutical agents (i.e., drugs) cannot be simply linked to the nanocrystal surface, because the combination of the nanocrystal surface and a number of other parameters governs their distribution and fate in the human body [16]. A sparingly soluble drug linked to the nanocrystal surface usually results in poor colloidal stability in the formulation, which is the first and most important reason for the lack of success in the formulation of such medicines. The challenges that are faced in the design of individual iron oxide nanocrystals are also related to the unsolvable difficulties associated with the loading of drugs into the nanocrystals’ interior. Also, for the development of a magnetically-responsive drug delivery system, the magnetic force exerted on an individual superparamagnetic iron oxide nanocrystal with a size <20 nm is not large enough to overcome the associated nanocrystal thermal fluctuations or Brownian motion, even in strong magnetic field gradients [17].

These shortcomings dictate the design of any magnetic drug-delivery systems where magnetic multi-core nanocrystal clusters (i.e., magnetic nanocarriers) offer an effective alternative to individual iron oxide nanocrystals in magnetic drug delivery [17]. The controlled assembly of a number of small superparamagnetic nanocrystals (of <20 nm) into multi-core clusters (of >50 nm) preserves their superparamagnetism and significantly increases their translational movement in a magnetic field gradient, which is a prerequisite for magnetically-responsive drug delivery [18]. The size of the final drug-loaded carrier is of primary importance for parenteral administration; it should be below a few hundred nanometers, although larger sizes of magnetic carriers result in more rapid magnetic responsiveness.

Versatile magnetically-responsive platforms of multi-core particles have been developed over recent decades, including: (i) magnetic microbeads, where magnetic nanocrystals are synthesized inside polymer or mesoporous silica microspheres [19]; (ii) magnetic nanocarriers, where small multi-core nanocrystal clusters are initially assembled, and then coated with mesoporous silica [20,21,22,23]; (iii) magnetic liposomes, where magnetic nanocrystals are either loaded into the hydrophilic liposome interior or are incorporated into the lipid bilayer [24,25,26,27,28]; and (iv) composite magnetic nanocarriers, where a number of nanocrystals are co-assembled with biodegradable polymers or lipids, to form nanoscale spheres [29].

The design of multi-core nanocrystal assemblies allows spatially-controlled drug loading. A drug can be effectively loaded either into the multi-core particle interior, inside a mesoporous silica shell, or either into the hydrophilic liposome interior, or into its lipid bilayer [17]. The physicochemical properties of the drug and its compatibility with the components of the multi-core nanocarrier dictate the possibility of effectively loading the drug into the nanocarrier. For drug loading into a lipid or polymer multi-core matrix, the drug should be relatively hydrophobic and compatible with the lipids or polymers, and it should not impair the colloidal stability of the admixed magnetic nanocrystals during the formulation.

Magnetic liposomes have been intensively studied as magnetic nanocarriers for drug delivery due to their relatively easy and low-cost preparation. Relatively hydrophobic and water-soluble substances can be incorporated. The amount of drug to be loaded into a hydrophobic bilayer is relatively small, while on the other hand, a water-soluble drug can be loaded into the aqueous liposome interior in relatively large amounts. However, nanocarriers are usually developed for sparingly soluble drug molecules [30,31].

Rodrigues et al. (2017) synthesized magnetic manganese ferrite liposomes and loaded them with thieno(3,2-b)pyridine-7-arylamines, as promising antitumor compounds [30]. The loading efficiency of the compounds was >75%, and the achieved growth inhibitory effect of such loaded magnetic liposomes on tumor cell lines showed the feasibility of using such a nanocarrier system to successfully deliver drugs to desired target tissues and cells. Nobuto et al. (2004) prepared magnetic liposomes loaded with doxorubicin; these were used to treat osteosarcoma in hamsters, with exposure to an external magnetic field used to concentrate the magnetic liposomes in the tumorous tissue [25]. They confirmed that this magnetic drug targeting increased the antitumor effects compared to the control. Mikhaylov et al. (2011) used magnetic ferri-liposomes loaded with doxorubicin and the cysteine cathepsin inhibitor JPM-565 for drug delivery and simultaneous NMR imaging of their distribution in the tumorous tissue [26]. These magnetically-targeted doxorubicin-loaded nanocarriers reduced the tumor size by 90%, while only about 60% tumor reduction was shown in the control group treated with doxorubicin without the magnetic nanocrystals. Moreover, it was confirmed that JPM-565 inhibited cysteine cathepsin significantly when delivered by the magnetic nanocrystals. Hardiansyah et al. (2017) loaded a hydrophobic model drug, curcumin, into polyethylene glycol (PEG)-ylated magnetic liposomes and tested this nanocarrier on fibroblasts and MCF-7 cells [27]. They studied the magnetic hyperthermia-triggered drug release in vitro, and the cellular internalization and toxicity of the liposomes induced by their magnetic guidance. The accumulation of these magnetic liposomes in the target cells was enhanced when an external magnetic field gradient was used. Rapid release of the loaded curcumin was induced by magnetic heating to 45 °C, while, in contrast, no significant release was detected without magnetic heating to 37 °C. They showed that these curcumin-loaded magnetic liposomes resulted in the death of the MCF-7 cells in vitro. Toro-Cordova et al. (2018) used PEGylated magnetic liposomes loaded with cisplatin and an external magnetic field to guide their magnetic liposomes into HeLa cells, where they successfully triggered apoptosis [28]. Although liposomes offer many of the above-described advantages, the main disadvantages are their physical and/or chemical instability [32,33], low drug loading or encapsulation efficiency [33], poor repeatability in their preparation [33] and difficulties with sterilization of formulations containing liposomes [33].

Magnetic-composite nanocarriers represent a good alternative to core-shell nanostructures and magnetic liposomes. These have relatively large cargo capacities and better physical stability compared to magnetic liposomes. They are also suitable to carry relatively hydrophobic drugs, and most importantly, they have simple preparation methods. The biocompatibility of such composite nanocarriers depends on the materials used for their preparation and their surface modification [17]. Magnetic nanocrystals can form composites with biodegradable polymers, such as poly-lactic-co-glycolic acid, or lipids, such as glyceryl behenate, which can result in polymer or solid lipid magnetic nanocarriers, respectively. Park et al. (2016) prepared magnetic nanocarriers that were composed of poly-lactic-co-glycolic acid, 5-nitrobenzoxadiazolyl-labelled cholesterol, and polydopamine-modified magnetic nanocrystals [34]. The bio-distribution of these composite nanocarriers showed increased accumulation in tumorous tissue due to the enhanced permeability and retention effects. However, greater accumulation in the targeted tissues was achieved when an external magnetic field gradient was applied. Hosseinzadeh et al. (2019) prepared chitosan-based magnetic nanocarriers, and studied their controlled release of doxorubicin at different pHs and temperatures in vitro [35]. Generally, doxorubicin release increased with decreasing pH of the medium (from 7.4 to 2.0) and with increasing temperature (from 25 °C to 45 °C). Oliveira et al. (2018) prepared solid lipid nanocarriers that were loaded with paclitaxel, L-α-phosphatidylcholine, glyceryl monostearate, and magnetic nanocrystals, and studied the release of paclitaxel by pulsed magnetic hyperthermia in vitro [36]. The magnetic hyperthermia triggered the release of paclitaxel from the nanocarriers, whereby total drug release increased from 10% to 85% in 24 h.

**Figure 1:**
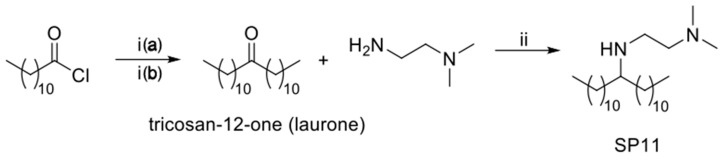
(i) (**a**) Et_3_N, diethyl ether, 0 °C, 1 h; (**b**) 2% KOH (aq); (ii) NaB(AcO)_3_H, AcOH, 1,2-dichloroethane RT, 15 h.

**Figure 2:**
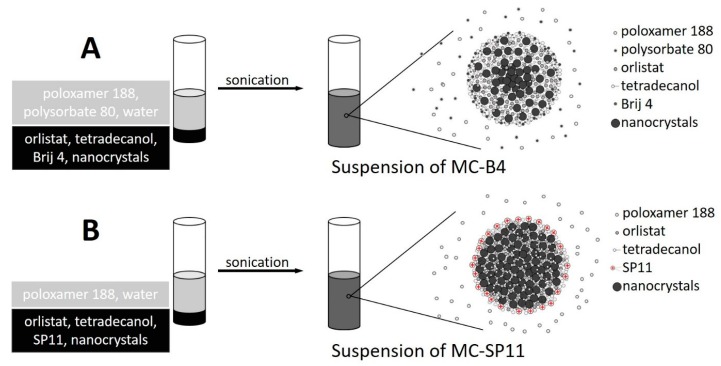
Schematic representation of the MC-B4 (**A**) and MC-SP11 (**B**) magnetic nanocarriers preparation method.

In general, a drug can be loaded into magnetic composite nanocarriers simply by mixing it with other hydrophobic components during a one-pot preparation method. Here, we present a novel one-pot method for the preparation of magnetic composite nanocarriers that are loaded with orlistat, a model drug with potential anticancer activity [37]. Orlistat was reported to have fatty acid synthase (FASN) inhibitory effect. FASN is overexpressed in several human carcinomas. The preparation method is solvent-free and based on hot homogenization of the hydrophobic phase, which contained the commercial surfactant polyethylene glycol dodecyl ether (Brij 4) or our prepared *N*^1^,*N*^1^-dimethyl-*N*^2^-(tricosan-12-yl)ethane-1,2-diamine (SP11) (the synthesis protocol of SP11 is shown in Figure 1), into aqueous phase that contained commercial surfactants: poloxamer 188 and polysorbate 80 or poloxamer 188 only, respectively (Figure 2). The orlistat loading and release from the nanocarriers were investigated. These data show that the surfactants used in the preparation of nanocarriers have significant effects on the control of the nanocarrier morphology and their drug release kinetics.

## 2. Materials and Methods

### 2.1. Chemicals

All chemicals used were of reagent grade and from commercial sources. The magnetic nanocrystals (mean diameter, 10 nm; iron-oxide content, 80–85 wt.%; saturation magnetization, Ms ~65 Am^2^/kg γ-Fe_2_O_3_) were kindly provided by Nanos Scientificae Ltd. (Nanos SCI, Ljubljana, Slovenia). Ethanol (96%) was from Pharmachem (Sušnik Jožef, Ljubljana, Slovenia), dichloromethane was from Honeywell (Riedel-de Haen, Seelze, Germany), [(2S)-1-[(2S,3S)-3-hexyl-4-oxooxetan-2-yl] tridecan-2-yl] (2S)-2-formamido-4-methylpentanoate (orlistat) was from Ranbaxy (Gurgaon, Sinnar, India), 4-(2-hydroxyethyl)-1-piperazineethanesulfonic acid and concentrated hydrochloric acid (HCl) were from Sigma Aldrich (St. Louis, MO, USA), tetradecan-1-ol (tetradecanol) was from Acros Organics (Geel, Belgium), polyethylene glycol dodecyl ether (Brij^®^ L4, Brij 4) was from Sigma Aldrich (St. Louis, MO, USA), polyoxyethylene (20) sorbitan monooleate (Tween^®^ 80; polysorbate 80) was from Merck KGaA (Darmstadt, Germany), and nonionic poly (ethylene oxide)-poly (propylene oxide) copolymer (Lutrol^®^ F-68; poloxamer 188) was from Sigma-Aldrich Chemie GmbH (Steinheim, Germany). *N*^1^,*N*^1^-dimethyl-*N*^2^-(tricosan-12-yl)ethane-1,2-diamine (SP11) was synthesized in our laboratory. The purified water used in this study was prepared by reverse osmosis at the Faculty of Pharmacology, University of Ljubljana and at the Jožef Stefan Institute.

### 2.2. Methods

#### 2.2.1. Synthesis of *N*^1^,*N*^1^-dimethyl-*N*^2^-(tricosan-12-yl)ethane-1,2-diamine (SP11)

##### Synthesis of Tricosan-12-one (Laurone)

The synthesis of tricosan-12-one was carried out as reported by Sauer (2003) [38]. The detailed synthesis is given in Supporting Information S1.

##### Synthesis of *N*^1^,*N*^1^-dimethyl-*N*^2^-(tricosan-12-yl)ethane-1,2-diamine (SP11)

To a solution of laurone (500 mg, 1.48 mmol, 1.0 equiv.) in 1,2-dichloroethane (5.5 mL), *N*^1^,*N*^1^-dimethylethane-1,2-diamine (260 mg, 2.96 mmol, 2.0 equiv.) and NaB(AcO)_3_H (627 mg, 2.96 mmol, 2 equiv.) were added. After 1 h, acetic acid (178 μL, 3.11 mmol, 2.1 equiv.) was added, and the reaction mixture was left to react for 15 h. The reaction mixture was then diluted with CH_2_Cl_2_ (30 mL), and the organic phase was washed with 1 M aqueous NaOH solution (3 × 15 mL) and brine (30 mL), and dried over Na_2_SO_4_. The solvent was evaporated under reduced pressure. The crude product was purified by flash chromatography on silica gel (dichloromethane:MeOH:Et_3_N, 90:10:1), to give the desired product (79%) as a colorless oily liquid. ^1^H NMR (400 MHz, CDCl_3_): δ (ppm) 3.58 (bs, 1H), 2.71 (t, J = 6.4 Hz, 2H), 2.52 (quint, J = 6.0 Hz, 1H), 2.46 (t, J = 6.4 Hz, 2H), 2.22 (s, 6H), 1.0–1.5 (m, 40H), 0.87 (t, J = 6.8 Hz, 6H). ^13^C NMR (101 MHz, CDCl_3_) δ (ppm) 58.43, 58.11, 45.44, 43.98, 33.47, 32.05, 29.98, 29.79, 29.77, 29.76, 29.75, 29.48, 25.83, 22.82, 14.26. HRMS (ESI), m/z calcd for C_27_H_59_N_2_ 411.4673 (M + H)^+^, found 411.4671.

#### 2.2.2. One-Pot Preparation of Magnetic Nanocarriers with Brij 4 (MC-B4)

Although this preparation method is one-pot, the procedures are complex, and are therefore composed of many consecutive steps. First, the homogeneous mixture of orlistat (5.0 mg), tetradecanol (27.5 mg), Brij 4 (18.8 µL), and the magnetic nanocrystals (21.0 mg) was prepared, and then heated to ~60 °C for 10 min. Separately, the aqueous phase was prepared by dissolving poloxamer 188 (60.0 mg) and polysorbate 80 (60 µL) in 60 mL distilled water. Once dissolved, half of the poloxamer 188-polysorbate 80 solution (30 mL) was thermostated at 4 °C, with the other half thermostated at 50 °C. Next, the hot homogenization step was performed by addition of the pre-heated aqueous phase to the hydrophobic phase, and the mixture was immediately sonicated while keeping the temperature constant at ~50 °C. The sonication (Sonics VibraCell VCX 500, model CV33) details were: amplitude, 20% (1.5 min; pulser 1 s ON/ 1 s OFF); amplitude 35% (2 min; pulser 1 s ON/ 1 s OFF). Then, the 30 mL of the cold poloxamer 188-polysorbate 80 solution was rapidly added to the mixture, to quickly reach room temperature for the final mixture. The sample was then thermostated at 4 °C for at least 1 h before being further processed. Finally, the magnetic MC-B4 nanocarriers produced were separated from the mixture by centrifugation (50,000× g, 5 min, 4 °C), dispersed in distilled water, and then stored at 8 °C.

#### 2.2.3. One-Pot Preparation of Magnetic Nanocarriers with SP11 (MC-SP11)

The method for the preparation of MC-SP11 was, in general, similar to that for MC-B4. First, the homogeneous mixture of orlistat (5.0 mg), tetradecanol (27.5 mg), SP11 (17.8 mg), and the magnetic nanocrystals (21.0 mg) was prepared, and heated to 80 °C for 10 min. Separately, the aqueous phase was prepared by dissolving poloxamer 188 (60.0 mg) in 60 mL distilled water and adjusting this to pH 3 with 0.1 M HCl. Half of the poloxamer 188 (0.1 wt.%) solution (30 mL) was thermostated at 4 °C, and the other half was thermostated at 75 °C. Next, the hot homogenization step was performed by addition of the pre-heated aqueous phase to the hydrophobic phase, and the mixture was immediately sonicated while the temperature was kept constant at ~75 °C. The sonication details were: amplitude, 20% (1.5 min; pulser 1 s ON/1 s OFF). Then, the 30 mL of cold poloxamer 188 solution was rapidly added to this mixture to rapidly solidify the droplets of the hydrophobic phase, due to the decrease in temperature of the final mixture. The sample was then thermostated at 4 °C for at least 1 h before being further processed. Finally, the magnetic MC-SP11 nanocarriers produced were separated from the mixture by centrifugation (50,000× g, 5 min, 4 °C), dispersed in distilled water, and then stored at 8 °C.

#### 2.2.4. Determination of Drug Loading

To determine the drug loading into the magnetic nanocarriers, 1 mL of the investigated nanocarrier dispersion was added to 10 mL 96% ethanol and sonicated for 30 min in an ultrasonic bath (Sonis 4; Iskra Pio, Slovenia). Next, the sample was incubated in a water bath at 50 °C for 24 h, with stirring (400 rpm), and then sonicated again in the ultrasonic bath for 15 min. Then 1 mL of sample was withdrawn and centrifuged (16,100× g, 10 min, 40 °C; Centrifuge 5415R; Eppendorf, Germany), to remove solid particles from the medium. Then 500 μL of the transparent supernatant was removed for further analysis by HPLC, to determine the drug concentration in the medium.

Equation (1) was used to calculate the drug loading as wt.% of the drug in the drug delivery system:

(1) $$\mathrm{drug}\;\mathrm{loading}\;(\%)=\frac{w_{\mathrm{drug}}}{w_{\mathrm{nc}}+w_{\mathrm{drug}}}\times 100\%,$$

where w_drug_ is the weight of drug in the dispersion of the drug-loaded nanocarriers, and w_nc_ is the weight of the drug-free nanocarriers.

#### 2.2.5. Drug-Release Studies

To determine the drug release from the orlistat-loaded magnetic nanoparticles, 1 mL of the investigated sample was transferred to 10 mL 5 mM HEPES (pH 5.3) and stirred in a water bath at 37 °C (400 rpm). At predetermined times (i.e., 2 h, 3 h, 24 h), 500 μL of sample was withdrawn and centrifuged (16,100× g, 37 °C, 10 min) without any replacement of the withdrawn medium. After centrifugation, 400 μL of the supernatant was removed for further analysis by HPLC, to determine the concentration of the released drug. Three replicates were performed, and the final data are expressed as the means of these. The cumulative drug release profile is expressed as the percentage of total drug released over time, based on the predetermined drug loading.

### 2.3. Characterization of Tricosan-12-One (Laurone) and SP11

Nuclear magnetic resonance spectra were measured on an NMR spectrometer (Avance III; Bruker, Billerica, MA, USA) using deuterated chloroform (CDCl_3_) as the solvent. The residual nondeuterated solvent peak was used as the internal standard. High resolution mass spectrometry (HRMS) measurements were performed on a mass spectrometer (QExactive Plus; Thermo Scientific, Waltham, MA, USA) with electrospray ionisation. The NMR spectra are given in Supporting Information S2 (Figures S1–S6), and the HRMS measurements are given in Supporting Information S3 (Figures S7 and S8).

### 2.4. HPLC Analysis of Orlistat

The concentrations of orlistat in the samples were determined using an HPLC system (Series 1100/1200; Agilent Technologies, Santa Clara, CA, USA) equipped with a UV-VIS detector and the ChemStation data-acquisition system. Chromatographic separation was performed on a reversed-phase column (Gemini-NX C18; 50 × 3.0 mm, 3 μm particle size; Phenomenex, Torrance, CA, USA) at 40 °C, using acetonitrile −0.1% orthophosphoric acid (70:30, v/v) as the mobile phase, at a flow rate of 1 mL/min. The injection volume was between 10 μL and 25 μL, depending on the sample type. Detection was carried out at a wavelength of 205 nm. A representative HPLC chromatogram of orlistat is given in Supporting Information S4 (Figure S9).

### 2.5. Characterization of the Magnetic Nanocarriers

The structure and morphology of the magnetic nanocarriers were examined by transmission electron microscopy (TEM; JEM 2100; JEOL, Akishima, Japan), operated at 200 kV. A drop of suspension was placed on the TEM grid and dried at room temperature. The mean particle size was determined based on an analysis of at least 100 particles. The mean hydrodynamic size of the magnetic nanocarriers was determined by photon correlation spectroscopy (Zetasizer Nano ZS; Malvern Instruments, Malvern, UK). The particle charge was quantified as the zeta potential by laser Doppler anemometry (Zetasizer Nano ZS; Malvern Instruments, Malvern, UK). Representative distributions of the zeta-potential and the intensity-weighted distributions of the hydrodynamic sizes for both of the sample types are given in Supporting Information S5 and S6 (Figures S10–S12), respectively. Magnetic measurements were performed using a vibrating-sample magnetometer (7400 series; Lake Shore Cryotronics, Westervile, OH, USA), with the maximum field of 10 kOe, at room temperature. Thermogravimetric analyses (TGA) were performed in the temperature range 30–500 °C in air with a heating rate of 10 °C/min, using a thermogravimeter Mettler-Toledo (TGA/DSC 1 Stare System). The concentration of the magnetic nanocarriers in the final water suspension was determined thermogravimetrically, based on drying 1 mL of sample at 80 °C overnight in a heated oven.

## 3. Results and Discussion

**Figure 3:**
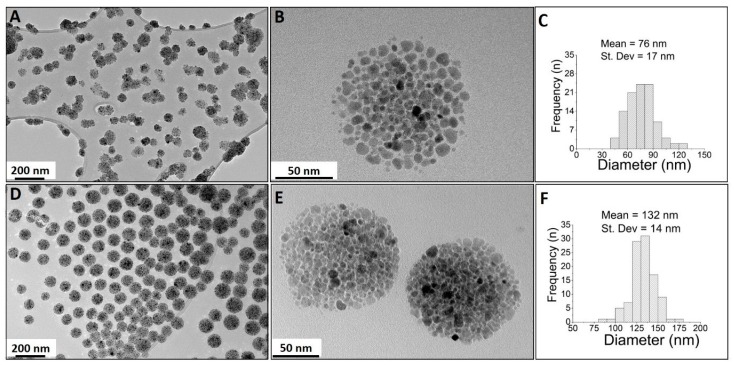
Transmission electron microscopy images of the magnetic multicore nanocarriers and their particle size distributions: MC-B4 (**A**–**C**) and MC-SP11 (**D**–**F**). Low (**A**,**D**) and high (**B**,**E**) magnification TEM images and nanocarrier size distributions (**C**,**F**) were determined based on the TEM images.

The key parameter for effective hydrophobic phase assembly into spherical droplets is the excellent colloidal stability of the magnetic nanocrystals in the hydrophobic mixture that contains the chemically diverse molecules. This results in the formation of monodispersed multi-core nanocarriers. Two different nanocarrier formulations were investigated, which differed in terms of both the hydrophobic and aqueous phases. TEM analyses revealed monodispersed spherical particles of sizes of 76 ± 17 nm for the MC-B4 formulation, and 132 ± 14 nm for the MC-SP11 formulation (Figure 3).

**Figure 4:**
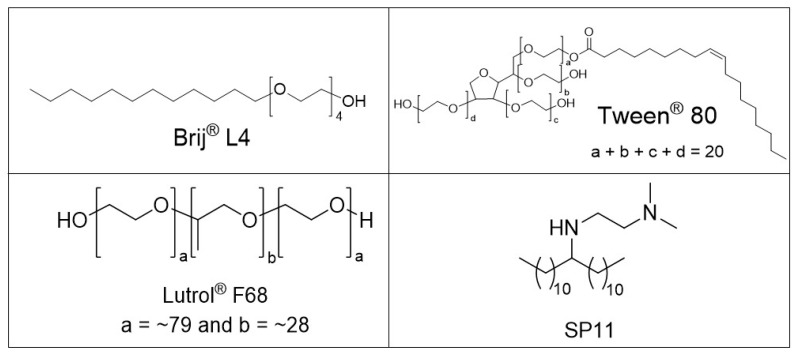
Chemical structures of the surfactants used in the preparation of the MC-B4 and MC-SP11 magnetic nanocarriers.

Transmission electron microscopy analysis at high magnification revealed the differences in surface morphology between these two multicore nanoparticle samples. The MC-B4 composite nanocarriers (Figure 3B) were composed of less tightly packed magnetic nanocrystals, compared to the more densely packed nanocrystals for the MC-SP11 nanocarriers (Figure 3E). The packing densities of the adjacent magnetic nanocrystals in the composite nanocarriers are related to differences in the nanocarrier composition or to the type of surfactants used in their formulations. As the initial composition of the hydrophobic phase (i.e., tetradecan-1-ol, superparamagnetic nanocrystals, orlistat) was identical for both the MC-B4 and MC-SP11 nanocarriers, the packing density can be related mainly to the type of surfactants used in their preparation. The newly-synthesized SP11 surfactant used for the preparation of the MC-SP11 nanocarriers appeared to be more effective for dispersal of the hydrophobic phase, compared to the combination of the nonpolar Brij 4 and polysorbate 80 in the MC-B4 formulation. Preparation of MC-B4 nanocarriers was not successful if SP11 was replaced with Brij 4 in hydrophobic phase while keeping all of the other components the same as for the MC-SP11 formulation. For the preparation of MC-B4 nanocarriers, the additional surfactant polysorbate 80 was needed in the aqueous phase for successful hot homogenization. The key difference in the structures of these surface-active compounds is the additional hydrocarbon tail in the SP11 molecule, compared to Brij 4 and polysorbate 80 (Figure 4). This will result in more hydrophobic characteristics for SP11. The two hydrocarbon tails of the SP11 molecule are responsible for the stronger association between SP11 and the hydrophobic components of the nanocarriers, which thus resulted in a more compact nanocrystals assembly for the MC-SP11 nanocarriers.

**Figure 5:**
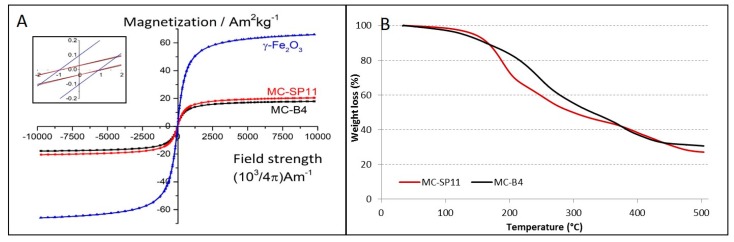
Magnetization as a function of magnetic field strength for the γ-Fe_2_O_3_ nanocrystals, MC-B4 and MC-SP11 nanocarriers (as indicated), measured at room-temperature (**A**). The coercive field is ~1 Oe for all three types of nanostructures (see inset) while remanent magnetizations are ~0.1 Am^2^/kg and ~0.03 Am^2^/kg for γ-Fe_2_O_3_ nanocrystals and both nanocarriers, respectively. TGA curves for the MC-B4 and MC-SP11 composite nanocarriers (**B**).

The magnetic behavior of the nanocarriers was investigated using a vibrating-sample magnetometer, at room temperature. Figure 5A shows the plots of magnetization versus applied magnetic field strength for both of the nanocarrier formulations. As can be seen, both of these delivery systems showed superparamagnetic behavior at ambient temperatures, as demonstrated by the symmetrical sigmoidal shapes of the magnetization curves and the absence of any hysteresis loops. The MC-B4 nanocarriers showed lower saturation magnetization (Ms ~17.9 Am^2^/kg) compared to the MC-SP11 nanocarriers (Ms ~20.4 Am^2^/kg) (Figure 5A). The hydrophobic phases of both of these nanocarrier formulations were optimized to contain the maximal amount of maghemite nanocrystals at fixed amounts of the other components (i.e., drug, tetradecanol, surfactants), to achieve the optimal magnetic responsiveness of the nanocarrier in the magnetic field gradient. Larger amounts of magnetic nanocrystals resulted in nonhomogeneous dispersion of the hydrophobic mixtures in the aqueous phase during the hot homogenization step. It appears that at certain loading levels of the hydrophobic phase with the magnetic nanocrystals, the hydrophobic mixture cannot be efficiently dispersed into small droplets, due to the changed rheological properties of the melted hydrophobic mixture (e.g., increased viscosity).

Based on the magnetic measurements, the estimated amounts of γ-Fe_2_O_3_ nanocrystals in the nanocarriers were 27.5 wt.% and 31.4 wt.% for the MC-B4 and MC-SP11 formulations, respectively. Similar values were determined by TGA which showed the remaining content of inorganic matter to be ~30.6 wt.% and ~27.1 wt.% for the MC-B4 and MC-SP11 formulations, respectively (Figure 5B). However, all these values are in good agreement with the initial composition of the hydrophobic phases used for the preparation of the MC-B4 and MC-SP11 nanocarriers (~29 wt.%). These data confirm the excellent colloidal stability of the hydrophobic phases before the hot homogenization. Generally, poor colloidal stability of magnetic nanocrystals in the hydrophobic phase is shown by visible nanocrystal flocculation, which usually results in an inhomogeneous composition of the resulting nanocarriers.

To test the versatility of the preparation method, only the drug (i.e., orlistat) was replaced with ibuprofen, and the physical stability of this new hydrophobic mixture was evaluated in comparison with the orlistat-containing hydrophobic phase. Both of these hydrophobic phases were placed above a permanent magnet (Q-50-25-10-LN; SuperMagnete, Gottmadingen Germany) for 15 min at 75 °C. The maghemite nanocrystals were partially separated out of the heated hydrophobic phase in the magnetic field gradient only with ibuprofen. This indicated the poor colloidal stability of the hydrophobic phase that contained ibuprofen, which might be related to the carboxyl functional group in the structure of the ibuprofen molecule. It is known that carboxyl-group-containing molecules interact with the surface of iron oxide nanocrystals. Indeed, in a study of ibuprofen-loaded nanoparticles that contained iron oxide, the formation of a new COO–Fe bond between the iron and the ibuprofen carboxyl group was shown by infrared spectroscopy at 1594 cm^−1^ [39]. Therefore, when carboxyl-group-containing molecules are in contact with iron oxides, they will be expected to impair the colloidal stability of iron oxide nanocrystals dispersed in a hydrophobic phase.

The hydrodynamic size measurements here revealed that the mean particle sizes for both of these formulations were <160 nm, and they did not change over at least two months. These MC-B4 and MC-SP11 nanocarriers had mean sizes of ~84 nm (polydispersivity index (PDI, 0.24) and ~154 nm (PDI, 0.45), respectively. The zeta potential measurements at pH 7.3 revealed significant differences for both of these formulations, i.e., −12.3 ± 1.8 mV and 42.3 ± 0.6 mV for the MC-B4 and MC-SP11 nanocarriers, respectively. This difference was related to the chemical properties of the surfactant molecules used (Figure 4). The MC-B4 nanocarriers were assembled using Brij 4 and polysorbate 80, which contain a single nonionic PEG chain and multiple PEG chains, respectively, as polar heads in the structure of surfactant. As PEG chains are nonionic, they stabilize the system only sterically, and do not significantly affect the electrostatic potential or nanocarrier surface. In contrast, the newly-synthesized surfactant SP11 was designed to affect the particle surface charge, and thus, the zeta potential, due to the presence of secondary and tertiary amine functional groups in the polar head of the molecule. The ionized amine groups resulted in positive zeta potential under acidic and neutral conditions, and therefore, provided electrostatic rather than steric stabilization of the dispersion of the MC-SP11 nanocarriers.

One of the most important characteristics of the nanoscale drug delivery system is its drug-loading capacity. The drug loading of the MC-B4 and MC-SP11 nanocarriers was 4.77 wt.% and 7.65 wt.%, respectively. These data are unexpected, because the magnetic measurements showed higher proportions of the organic phase (which contained tetradecanol, orlistat, Brij 4) in the MC-B4 nanocarriers compared to the MC-SP11 nanocarriers. This indicates that the organic phase of MC-B4 contained lower proportions of orlistat compared to the composition of the organic phase in MC-SP11. The MC-SP11 formulation thus demonstrated higher drug loading than that of MC-B4, probably due to relatively greater numbers of nanocrystals and their denser packing in the MC-SP11 nanocarriers, which might prevent premature drug release, and thus, drug loss during preparation of the nanocarriers. On the other hand, the smaller nanocrystal packing density for MC-B4 appears to be linked to higher proportions of the thermo-sensitive component, tetradecanol.

The drug loading of these formulations reached comparable values to previously published data for drug delivery systems based on pure lipids or mixtures of magnetic nanocrystals and lipids or polymers. For example, Delgado-Rosales et al. (2018) reported 8.9 wt.% and 8.4 wt.% drug loading for ibuprofen-loaded magnetic nanoparticles based on poly-lactic-co-glycolic acid and glyceryl behenate, respectively [40]. Brezaniova et al. (2016) prepared solid lipid nanoparticles that were composed of tetradecanol, polymer surfactant, and temoporfin as their model drug [41]. The composition of these nanoparticles was similar to the formulations of the present study, but without magnetic nanocrystals. They achieved 3.96 wt.% loading of temoporfin. The comparison of the present data with this other study confirmed that the drug loading of the formulations here was relatively high, especially taking into account that the formulations in the present study contained relatively high amounts of magnetic nanocrystals.

**Figure 6:**
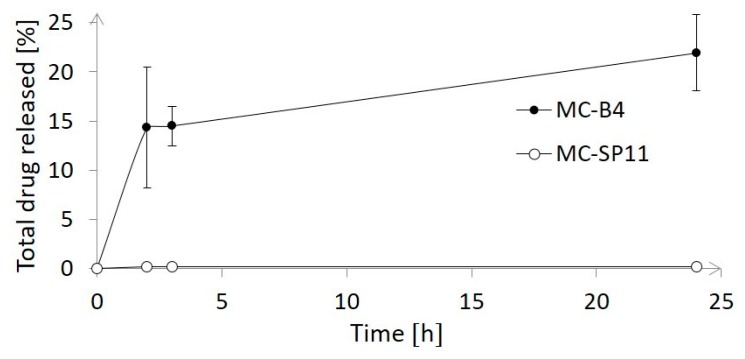
Drug release profiles for the MC-B4 and MC-SP11 nanocarriers at 37 °C.

The drug release studies performed in dissolution medium with weakly acidic pH, which mimic low pH in tumor microenvironment, revealed that ~22% of orlistat was released within 24 h from the MC-B4 nanocarriers, whereas no significant drug release was detected for the MC-SP11 nanocarriers over the same time period (Figure 6). These data are in good agreement with the hypothesis that orlistat is well entrapped in the MC-SP11 nanocarriers, and thus, cannot be released under the conditions used in the drug-release assay here. The investigations thus confirmed prolonged drug release only from the MC-B4 nanocarriers. These data are in line with previously published studies. Brezaniova et al. (2016) showed that <30% of the drug was released in 24 h from solid lipid nanoparticles composed of tetradecanol, polymer surfactant, and temoporfin as the model drug [33], which is comparable to the present formulation. As both of the present formulations contained tetradecanol, which is liquid above ~38 °C, temperature-triggered drug release is expected; this will be investigated in the future. This is important, especially for the MC-SP11 formulation, where no drug release was detected over 24 h. Thus, the drug release might be effectively triggered either by internal heating via magnetic hyperthermia or photothermia, or by the temperature difference between cancerous and normal tissues. The local increase in the temperature was expected to cause nanocarrier disintegration and rapid drug release. Temperature-triggered drug release studies exceed the framework of the present study, but will be investigated in detail in the near future.

## 4. Conclusions

Two magnetic nanocarrier formulations that differed in their surfactants and were loaded with orlistat were successfully developed using one-pot preparation method. This method is based on hot homogenization of the hydrophobic phase that contains nonpolar surfactant into the aqueous phase by ultrasonication. Two different nonpolar surfactants were studied for the preparation of the magnetic nanocarriers, as Brij 4 and our own SP11, for the MC-B4 and MC-SP11 nanocarriers, respectively. These nanocarriers were therefore composed of tetradecanol, superparamagnetic nanocrystals, orlistat, and surfactant. The formulations showed significantly different orlistat release kinetics, which were controlled solely by the surfactants. Prolonged drug release was seen for the MC-B4 nanocarriers, while no drug release was seen for the MC-SP11 nanocarriers under the same conditions. The magnetic nanocarriers were spherical in shape with TEM-determined mean particle sizes between 70 nm and 140 nm, high drug loading (4.77 wt.% to 7.65 wt.%) and good colloidal stability.

## Supplementary Materials

The following are available online at http://www.mdpi.com/1996-1944/12/3/540/s1. Figure S1: ^1^H NMR spectrum of laurone (12 ppm to −1 ppm), Figure S2: ^1^H NMR spectrum of laurone (4 ppm to 0.5 ppm), Figure S3: ^1^H NMR spectrum of SP11 (12 ppm to −1 ppm), Figure S4: ^1^H NMR spectrum of SP11 (4 ppm to 0.5 ppm), Figure S5: ^13^C NMR spectrum of SP11 (180 ppm to −10 ppm), Figure S6: ^13^C NMR spectrum of SP11 (80 ppm to 10 ppm), Figure S7: Reported HRMS measurement values, Figure S8: Calculated chemical formula, exact mass and molecular weight of SP11, Figure S9: Representative HPLC chromatograph of orlistat, Figure S10: Representative zeta-potential distributions for the formulations MC-B4 and MC-SP11, Figure S11: Logarithmic values of the intensity-weighted hydrodynamic size distributions for MC-B4, Figure S12: Logarithmic values of the intensity-weighted hydrodynamic size distributions for MC-SP11.

## References

[1] Lisjak D., Mertelj A. (2018). Anisotropic magnetic nanoparticles: A review of their properties, syntheses and potential applications. Prog. Mater. Sci..

[2] Kolosnjaj-Tabi J., Wilhelm C. (2017). Magnetic nanoparticles in cancer therapy: How can thermal approaches help?. Nanomedicine.

[3] Kavre I., Kostevc G., Kralj S., Vilfan A., Babič D. (2014). Fabrication of magneto-responsive microgears based on magnetic nanoparticle embedded PDMS. RSC Adv..

[4] Wells C., Vollin-Bringel O., Fiegel V., Harlepp S., Van der Schueren B., Bégin-Colin S., Bégin D., Mertz D. (2018). Engineering of Mesoporous Silica Coated Carbon-Based Materials Optimized for an Ultrahigh Doxorubicin Payload and a Drug Release Activated by pH, T, and NIR-light. Adv. Funct. Mater..

[5] Fiegel V., Harlepp S., Begin-Colin S., Begin D., Mertz D. (2018). Design of Protein-Coated Carbon Nanotubes Loaded with Hydrophobic Drugs through Sacrificial Templating of Mesoporous Silica Shells. Chem. A Eur. J..

[6] Kostevšek N., Abramovič I., Hudoklin S., Kreft M.E., Serša I., Sepe A., Vidmar J., Šturm S., Spreitzer M., Ščančar J. (2018). Hybrid FePt/SiO_2_/Au nanoparticles as a theranostic tool: In vitro photo-thermal treatment and MRI imaging. Nanoscale.

[7] Kolosnjaj Tabi J., Lartigue L., Javed Y., Luciani N., Pellegrino T., Wilhelm C., Alloyeau D., Gazeau F. (2016). Biotransformations of magnetic nanoparticles in the body. Nanotoday.

[8] Yarjanli Z., Ghaedi K., Esmaeili A., Rahgozar S., Zarrabi A. (2017). Iron oxide nanoparticles may damage to the neural tissue through iron accumulation, oxidative stress, and protein aggregation. BMC Neurosci..

[9] Malvindi M.A., De Matteis V., Galeone A., Brunetti V., Anyfantis G.C., Athanassiou A., Cingolani R., Pompa P.P. (2014). Toxicity assessment of silica coated iron oxide nanoparticles and biocompatibility improvement by surface engineering. PLoS ONE.

[10] Maier-Hauff K., Ulrich F., Nestler D., Niehoff H., Wust P., Thiesen B., Orawa H., Budach V., Jordan A. (2011). Efficacy and safety of intratumoral thermotherapy using magnetic iron-oxide nanoparticles combined with external beam radiotherapy on patients with recurrent glioblastoma multiforme. J. Neurooncol..

[11] Espinosa A., Kolosnjaj-Tabi J., Abou-Hassan A., Plan Sangnier A., Curcio A., Silva A.K.A., Di Corato R., Neveu S., Pellegrino T., Liz-Marzán L.M. (2018). Magnetic (Hyper)Thermia or Photothermia? Progressive Comparison of Iron Oxide and Gold Nanoparticles Heating in Water, in Cells, and In Vivo. Adv. Funct. Mater..

[12] Blanco-Andujar C., Walter A., Cotin G., Bordeianu C., Mertz D., Felder-Flesch D., Begin-Colin S. (2016). Design of iron oxide-based nanoparticles for MRI and magnetic hyperthermia. Nanomedicine.

[13] Hemsley B., Rollo M., Georgiou A., Balandin S., Hill S. (2018). The health literacy demands of electronic personal health records (e-PHRs): An integrative review to inform future inclusive research. Patient Educ. Couns..

[14] Espinosa A., Di Corato R., Kolosnjaj-Tabi J., Flaud P., Pellegrino T., Wilhelm C. (2016). Duality of Iron Oxide Nanoparticles in Cancer Therapy: Amplification of Heating Efficiency by Magnetic Hyperthermia and Photothermal Bimodal Treatment. ACS Nano.

[15] Kolosnjaj-Tabi J., Di Corato R., Lartigue L., Marangon I., Guardia P., Silva A.K.A., Luciani N., Clément O., Flaud P., Singh J.V. (2014). Heat-generating iron oxide nanocubes: Subtle “destructurators” of the tumoral microenvironment. ACS Nano.

[16] Ménard M., Meyer F., Parkhomenko K., Leuvrey C., Francius G., Bégin-Colin S., Mertz D. (2019). Mesoporous silica templated-albumin nanoparticles with high doxorubicin payload for drug delivery assessed with a 3-D tumor cell model. Biochim. Biophys. Acta Gen. Subj..

[17] Kralj S., Potrc T., Kocbek P., Makovec S.M. (2017). Design and Fabrication of Magnetically Responsive Nanocarriers for Drug Delivery. Curr. Med. Chem..

[18] Kralj S., Makovec D. (2014). The chemically directed assembly of nanoparticle clusters from superparamagnetic iron-oxide nanoparticles. RSC Adv..

[19] Philippova O., Barabanova A., Molchanov V., Khokhlov A. (2011). Magnetic polymer beads: Recent trends and developments in synthetic design and applications. Eur. Polym. J..

[20] Kralj S., Makovec D. (2015). Magnetic Assembly of Superparamagnetic Iron Oxide Nanoparticle Clusters into Nanochains and Nanobundles. ACS Nano.

[21] Gai S., Yang P., Ma P., Wang D., Li C., Li X., Niu N., Lin J. (2011). Fibrous-structured magnetic and mesoporous Fe_3_O_4_/silica microspheres: Synthesis and intracellular doxorubicin delivery. J. Mater. Chem..

[22] Tadic M., Kralj S., Jagodic M., Hanzel D., Makovec D. (2014). Magnetic properties of novel superparamagnetic iron oxide nanoclusters and their peculiarity under annealing treatment. Appl. Surf. Sci..

[23] Xiong L., Bi J., Tang Y., Qiao S.Z. (2016). Magnetic Core-Shell Silica Nanoparticles with Large Radial Mesopores for siRNA Delivery. Small.

[24] Rodrigues A.R.O., Ramos J.M.F., Gomes I.T., Almeida B.G., Araújo J.P., Queiroz M.J.R.P., Coutinho P.J.G., Castanheira E.M.S. (2016). Magnetoliposomes based on manganese ferrite nanoparticles as nanocarriers for antitumor drugs. RSC Adv..

[25] Nobuto H., Sugita T., Kubo T., Shimose S., Yasunaga Y., Murakami T., Ochi M. (2004). Evaluation of systemic chemotherapy with magnetic liposomal doxorubicin and a dipole external electromagnet. Int. J. Cancer.

[26] Mikhaylov G., Mikac U., Magaeva A.A., Itin V.I., Naiden E.P., Psakhye I., Babes L., Reinheckel T., Peters C., Zeiser R. (2011). Ferri-liposomes as an MRI-visible drug-delivery system for targeting tumours and their microenvironment. Nat. Nanotechnol..

[27] Hardiansyah A., Yang M.C., Liu T.Y., Kuo C.Y., Huang L.Y., Chan T.Y. (2017). Hydrophobic Drug-Loaded PEGylated Magnetic Liposomes for Drug-Controlled Release. Nanoscale Res. Lett..

[28] Toro-Cordova A., Flores-Cruz M., Santoyo-Salazar J., Carrillo-Nava E., Jurado R., Figueroa-Rodriguez P.A., Lopez-Sanchez P., Medina L.A., Garcia-Lopez P. (2018). Liposomes loaded with cisplatin and magnetic nanoparticles: Physicochemical characterization, pharmacokinetics, and in-vitro efficacy. Molecules.

[29] Kocbek P., Kralj S., Kreft M.E., Kristl J. (2013). Targeting intracellular compartments by magnetic polymeric nanoparticles. Eur. J. Pharm. Sci..

[30] Rodrigues A.R.O., Almeida B.G., Rodrigues J.M., Queiroz M.J.R.P., Calhelha R.C., Ferreira I.C.F.R., Pires A., Pereira A.M., Araújo J.P., Coutinho P.J.G. (2017). Magnetoliposomes as carriers for promising antitumor thieno[3,2-b]pyridin-7-arylamines: photophysical and biological studies. RSC Adv..

[31] Plassat V., Wilhelm C., Marsaud V., Menager C., Gazeau F., Renoir J.-M., Lesieur S. (2010). Anti-estrogen-loaded superparamagnetic liposomes for intracellular magnetic targeting and treatment of breast cancer tumors. Adv. Funct. Mater..

[32] Grit M., Crommelin D.J.A. (1993). Chemical stability of liposomes: implications for their physical stability. Chem. Phys. Lipids.

[33] Sharma A., Sharma U.S. (1997). Liposomes in drug delivery: progress and limitations. Int. J. Pharm..

[34] Park J., Kadasala N.R., Abouelmagd S.A., Castanares M.A., Collins D.S., Wei A., Yeo Y. (2016). Polymer-iron oxide composite nanoparticles for EPR-independent drug delivery. Biomaterials.

[35] Hosseinzadeh S., Hosseinzadeh H., Pashaei S., Khodaparast Z. (2019). Synthesis of stimuli-responsive chitosan nanocomposites via RAFT copolymerization for doxorubicin delivery. Int. J. Biol. Macromol..

[36] Oliveira R.R., Carrião M.S., Pacheco M.T., Branquinho L.C., de Souza A.L.R., Bakuzis A.F., Lima E.M. (2018). Triggered release of paclitaxel from magnetic solid lipid nanoparticles by magnetic hyperthermia. Mater. Sci. Eng. C.

[37] Mullen G.E., Yet L. (2015). Progress in the development of fatty acid synthase inhibitors as anticancer targets. Bioorganic Med. Chem. Lett..

[38] Sauer J.C. (2003). Laurone. Organic Syntheses.

[39] Huang S., Fan Y., Cheng Z., Kong D., Yang P., Quan Z., Zhang C., Lin J. (2009). Magnetic Mesoporous Silica Spheres for Drug Targeting and Controlled Release. J. Phys. Chem. C.

[40] Estefany Delgado-Rosales E., Quintanar D., Piñón-Segundo E., Magaña N., Leyva-Gómez G., Martínez-Martínez F., Mendoza Muñoz N. (2018). Novel drug delivery systems based on the encapsulation of superparamagnetic nanoparticles into lipid nanocomposites. J. Drug. Deliv. Sci. Technol..

[41] Brezaniova I., Hruby M., Kralova J., Kral V., Cernochova Z., Cernoch P., Slouf M., Kredatusova J., Stepanek P. (2016). Temoporfin-loaded 1-tetradecanol-based thermoresponsive solid lipid nanoparticles for photodynamic therapy. J. Control. Release.

