# Supplementary material for: One-Pot Method for Preparation of Magnetic Multi-Core Nanocarriers for Drug Delivery
Source: Materials (Basel). 2019 Feb 12;12(3):540. doi: 10.3390/ma12030540 (PMC6384742; doi:10.3390/ma12030540)
Supplement: Supplementary file 1 [file materials-12-00540-s001.pdf]

Supporting Information

One-Pot Method for Preparation of Magnetic Multi-Core Nanocarriers for Drug Delivery

Črt Dragar[1,2], Tanja Potrč[2], Sebastjan Nemec[1,2], Robert Roškar[2], Stane Pajk[2,3], Petra Kocbek[2], Slavko Kralj[1,2*]

1 Department for Materials Synthesis, Jožef Stefan Institute, Slovenia
2 Faculty of Pharmacy, University of Ljubljana, Slovenia
3 Laboratory of Biophysics, Jožef Stefan Institute, Slovenia

* Correspondence: slavko.kralj@ijs.si

Submitted for publication in
Materials

S1. Synthesis and characterization of N^1,N^1-dimethyl-N^2-(tricosan-12-yl)ethane-1,2-diamine - SP11

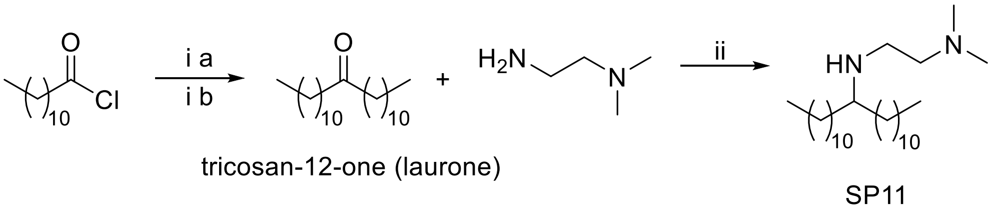

Scheme 1. (i) (a) Et_3N, diethyl ether, 0 °C, 1 h; (b) 2% KOH(aq); (ii) $NaB(AcO)_3H$, AcOH, 1,2-dichloroethane RT, 15 h.

S1.1. Synthesis of tricosan-12-one (laurone).

The synthesis of tricosan-12-one was carried out as reported by J. C. Sauer. (Sauer, J. C. (2003). Laurone. In Organic Syntheses, (Ed.). doi:10.1002/0471264180.os031.24). Lauroyl chloride (10 mL, 42 mmol, 1 equiv) was dissolved in diethyl ether (75 mL) and the solution was cooled on an ice bath. Et_3N (5.82 mL, 42 mmol, 1 equiv) was then added dropwise and the reaction mixture was stirred on an ice bath for one hour. The stirring was then discontinued and the reaction mixture was left at room temperature overnight. The reaction mixture was transferred to a separatory funnel, washed with 2 % sulfuric acid (2× 30 mL) and the solvents were removed under reduced pressure. 2 % KOH solution (60 mL) was added to the residue, the mixture was heated to the boiling point and then cooled on an ice bath. The waxy cake that formed on top of the aqueous layer was collected, washed with water and dried to yield the desired product (58 %) as a white solid.

S2. NMR data

Nuclear magnetic resonance spectra were measured on a Bruker Avance III NMR spectrometer at 400 MHz for proton spectra and at 101 MHz for carbon 13 spectra, using deuterated chloroform ($CDCl_3$) as solvent. As internal standard it was used the residual nondeuterated solvent peak (chloroform, $CHCl_3$).

S2.1. Proton NMR spectrum of laurone from 12 ppm to -1 ppm.

^{1}H NMR (400 MHz, $CDCl_3$): δ (ppm) 2.38 (t, J = 7.4 Hz, 4H), 1.63-1.47 (m, 4H), 1.37-1.17 (m, 32H), 0.88 (t, J = 6.7 Hz, 6H).

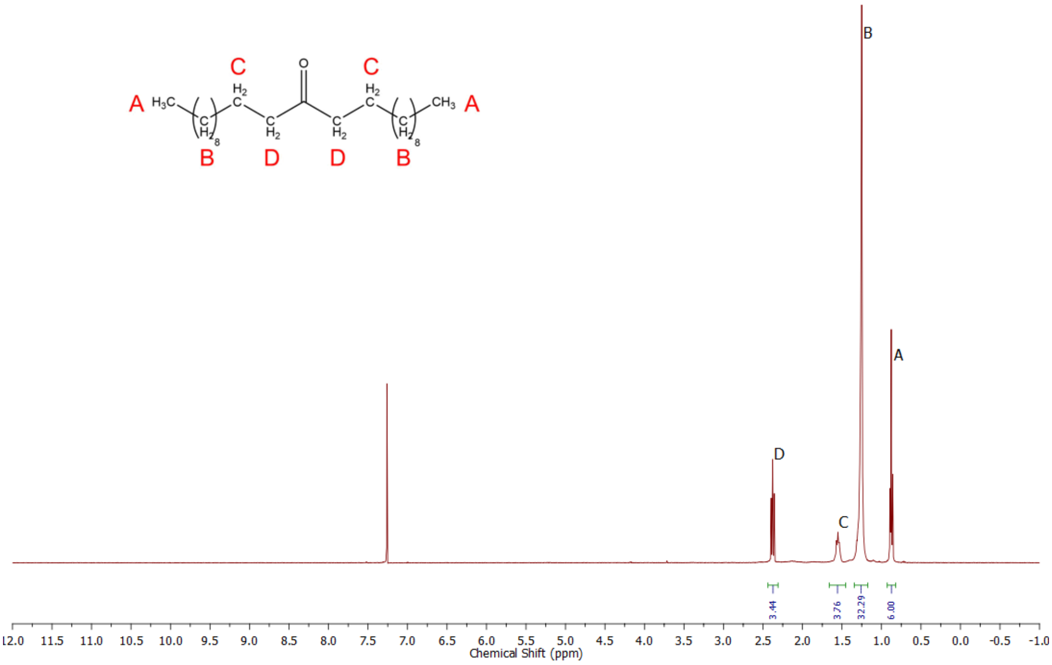

Figure S1: ^{1}H NMR spectrum (400 MHz, 298 K, **CDCl$_3$**) of laurone (12 ppm to -1 ppm).

S2.2. Proton NMR spectrum of laurone from 4 ppm to 0.5 ppm.

^{1}H NMR (400 MHz, CDCl$_3$): δ (ppm) 2.38 (t, J = 7.4 Hz, 4H), 1.63-1.47 (m, 4H), 1.37-1.17 (m, 32H), 0.88 (t, J = 6.7 Hz, 6H).

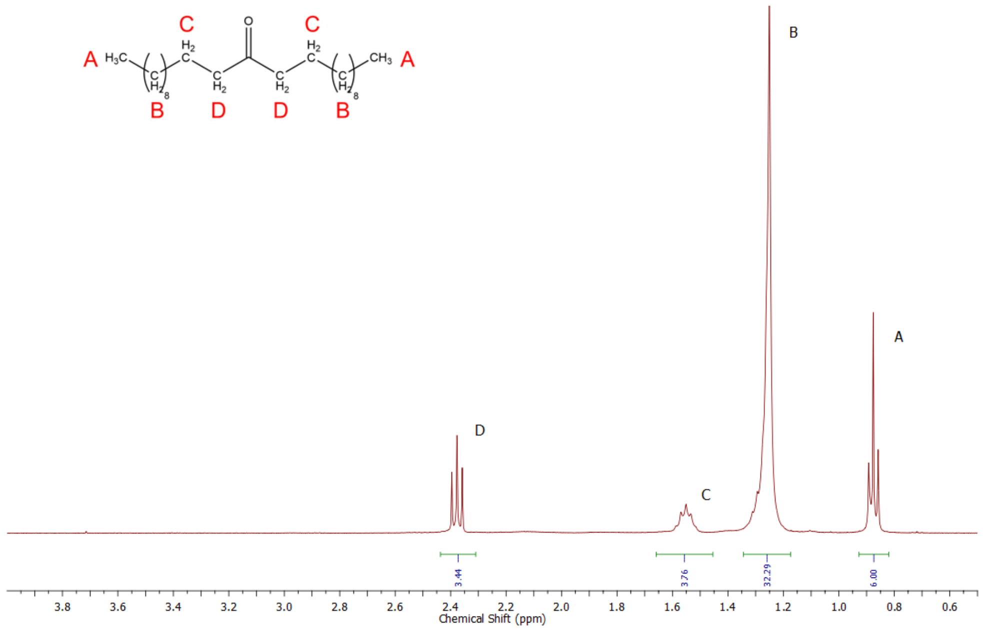

Figure S2: ^{1}H NMR spectrum (400 MHz, 298 K, **CDCl$_3$**) of laurone (4 ppm to 0.5 ppm).

S2.3 Proton NMR spectrum of SP11 from 12 ppm to -1 ppm.

^{1}H NMR (400 MHz, $CDCl_3$): δ (ppm) 3.58 (bs, 1H), 2.71 (t, J = 6.4 Hz, 2H), 2.52 (quint, J = 6.0 Hz, 1H), 2.46 (t, J = 6.4 Hz, 2H), 2.22 (s, 6H), 1.0-1.5 (m, 4H), 0.87 (t, J = 6.8 Hz, 6H).

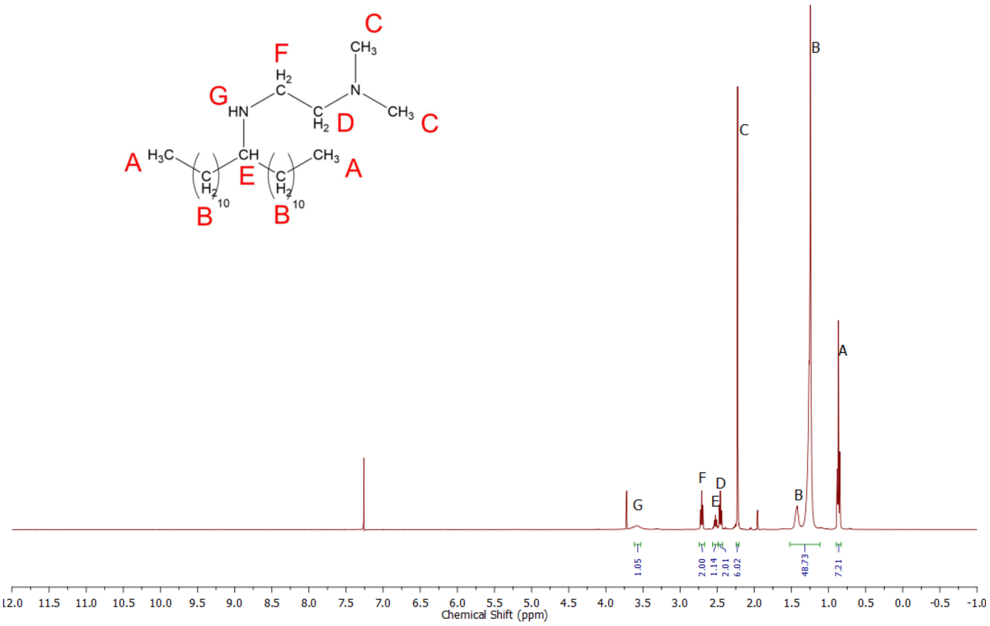

Figure S3: ^{1}H NMR spectrum (400 MHz, 298 K, $\mathbf{CDCl_3}$) of SP11 (12 ppm to -1 ppm).

S2.4 Proton NMR spectrum of SP11 from 4 ppm to 0.5 ppm.

^{1}H NMR (400 MHz, $CDCl_3$): δ (ppm) 3.58 (bs, 1H), 2.71 (t, J = 6.4 Hz, 2H), 2.52 (quint, J = 6.0 Hz, 1H), 2.46 (t, J = 6.4 Hz, 2H), 2.22 (s, 6H), 1.0-1.5 (m, 4H), 0.87 (t, J = 6.8 Hz, 6H).

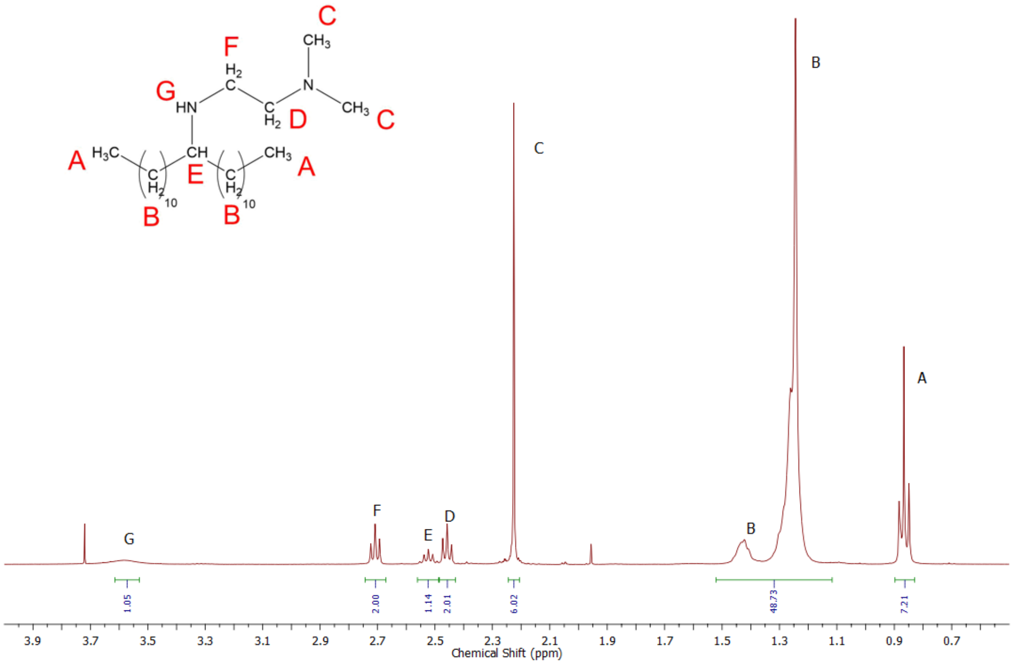

Figure S4: ^{1}H NMR spectrum (400 MHz, 298 K, **$CDCl_3$**) of SP11 (4 ppm to 0.5 ppm).

S2.5 Carbon NMR spectrum of SP11 from 180 ppm to -20 ppm.

^{13}C NMR (101 MHz, $CDCl_3$) δ (ppm) 58.43, 58.11, 45.44, 43.98, 33.47, 32.05, 29.98, 29.79, 29.77, 29.76, 29.75, 29.48, 25.83, 22.82, 14.26.

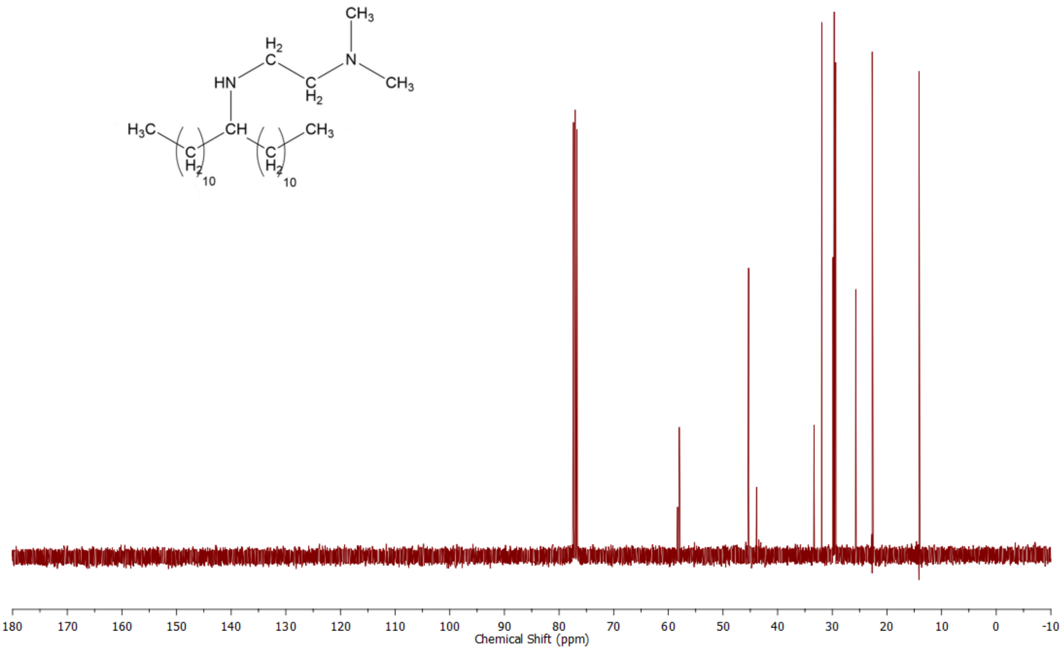

Figure S5: ^{13}C NMR spectrum (101 MHz, 298 K, **$CDCl_3$**) of SP11 (180 ppm to -10 ppm).

S2.6 Carbon NMR spectrum of SP11 from 65 ppm to 10 ppm.

^{13}C NMR (101 MHz, $CDCl_3$) δ (ppm) 58.43, 58.11, 45.44, 43.98, 33.47, 32.05, 29.98, 29.79, 29.77, 29.76, 29.75, 29.48, 25.83, 22.82, 14.26.

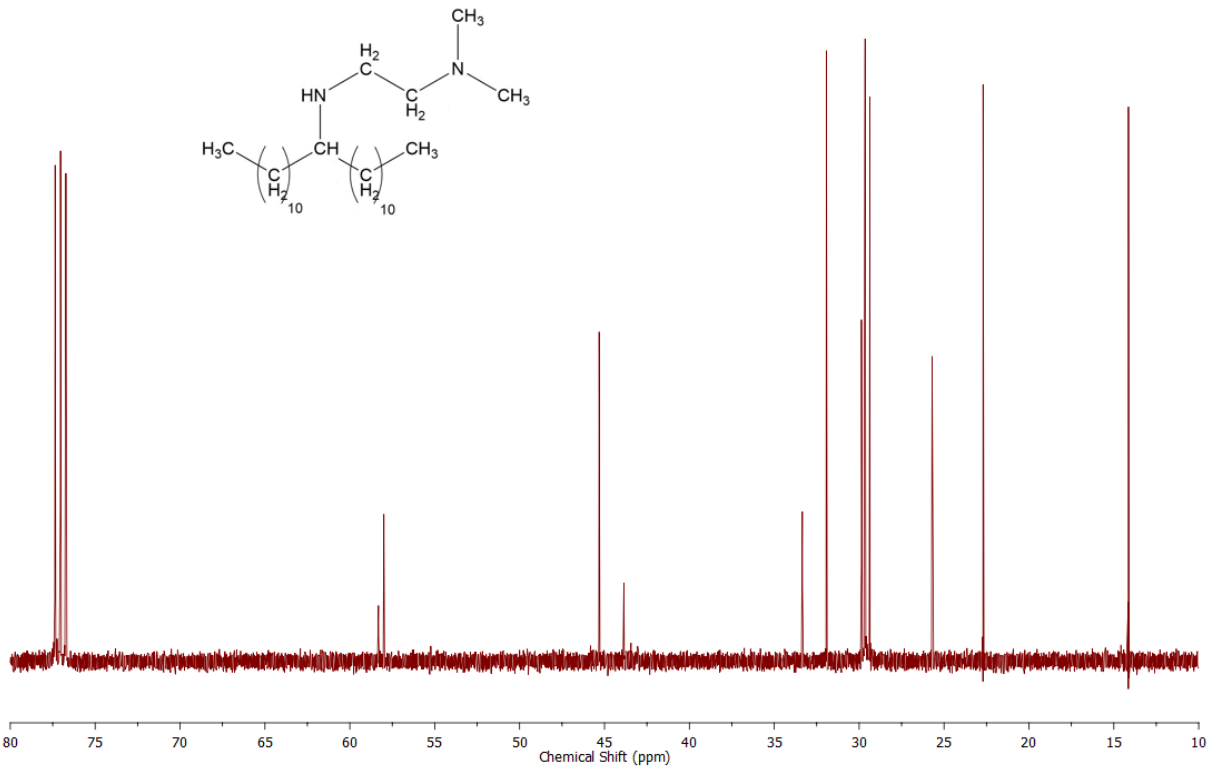

Figure S6: ^{13}C NMR spectrum (101 MHz, 298 K, **$CDCl_3$**) of SP11 (80 ppm to 10 ppm).

S3. High resolution mass spectrometry data

High resolution mass spectrometry measurements were performed on a Thermo Scientific QExactive Plus mass spectrometer with ESI ionisation.

S3.1 High resolution mass spectrometry measurement for N^1,N^1-dimethyl-N^2-(tricosan-12-yl)ethane-1,2-diamine (SP11)

HRMS (ESI), m/z calcd for $C_{27}H_{59}N_2$ 411.4673 $(M+H)^+$, found 411.4671.

Elemental composition search on mass 411.47

m/z= 406.47-416.47

m/z	Theo. Mass	Delta (ppm)	RDB equiv.	Composition
411.4671	411.4673	-0.53	-0.5	$C_{27}H_{59}N_2$

Figure S7: Reported HRMS measurement values.

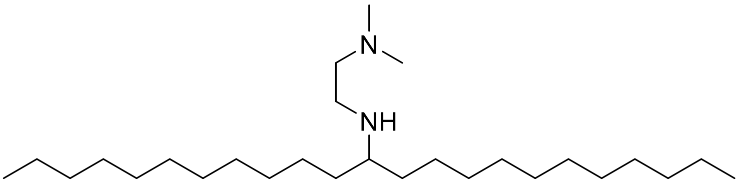

Figure S8: Calculated chemical formula, exact mass and molecular weight of SP11 using Perkin Elmer ChemDraw 16 Professional.

S4. Representative HPLC chromatograph of orlistat

For measuring concentrations of orlistat to determine the drug loading and drug release profile HPLC was used as described within methods. As shown in Figure S9 typical retentive time of orlistat is 4.390 minutes. Area under the curve or height of the peak is related to the concentration of orlistat, which enables calculating the concentration of it according to standard samples with known concentration.

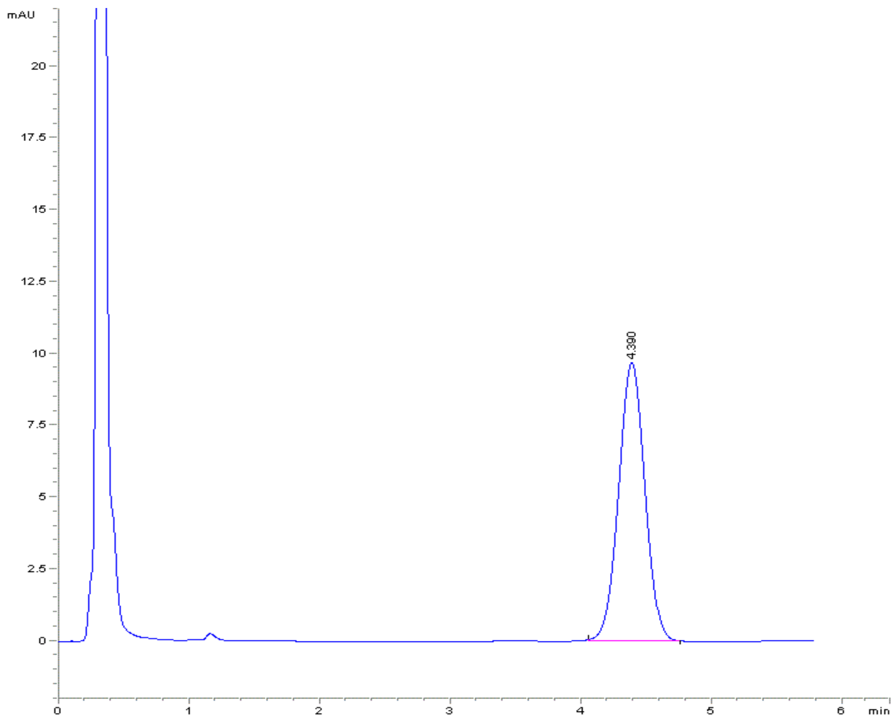

Figure S9: Representative HPLC chromatograph of orlistat.

S5. Zeta-potential measurements

Both magnetic nanocarrier suspensions were monitored with the measurements of their zeta-potentials by laser Doppler anemometry using the Zetasizer Nano ZS (Malvern Instruments, UK). The graphs in Figure S10 show representative distributions of apparent zeta-potentials.

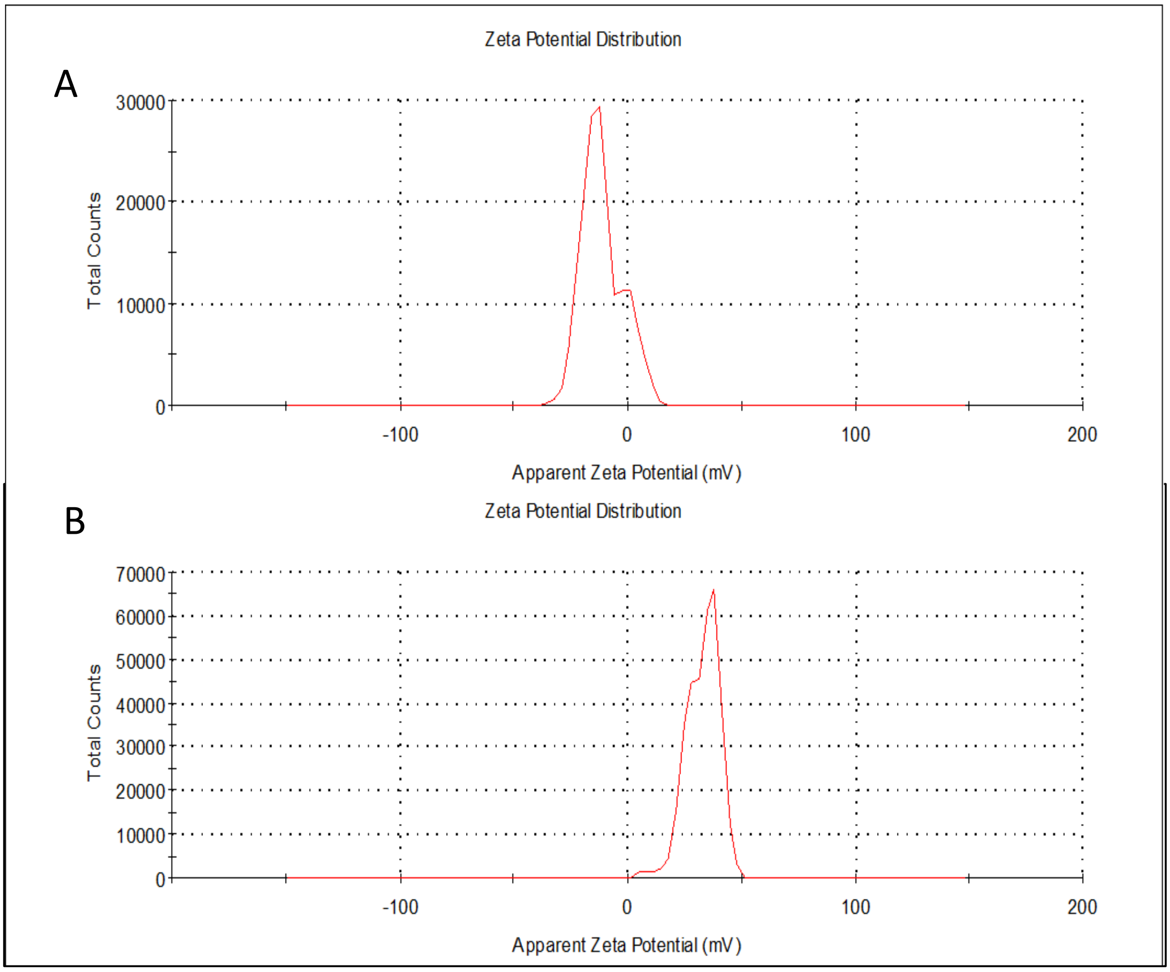

Figure S10: Representative zeta-potential distributions for the formulations MC-B4 (A) and MC-SP11 (B).

S6. Determination of the hydrodynamic sizes of magnetic nanocarriers

The determination of the hydrodynamic size of magnetic nanocarriers was performed by photon correlation spectroscopy using Zetasizer Nano ZS (Malvern Instruments, UK). Each individual sample was measured three-times and the corresponding standard deviations (SD) were calculated. The intensity-weighted distributions of the hydrodynamic sizes including SDs for both samples are shown in Figure S11 and Figure S12.

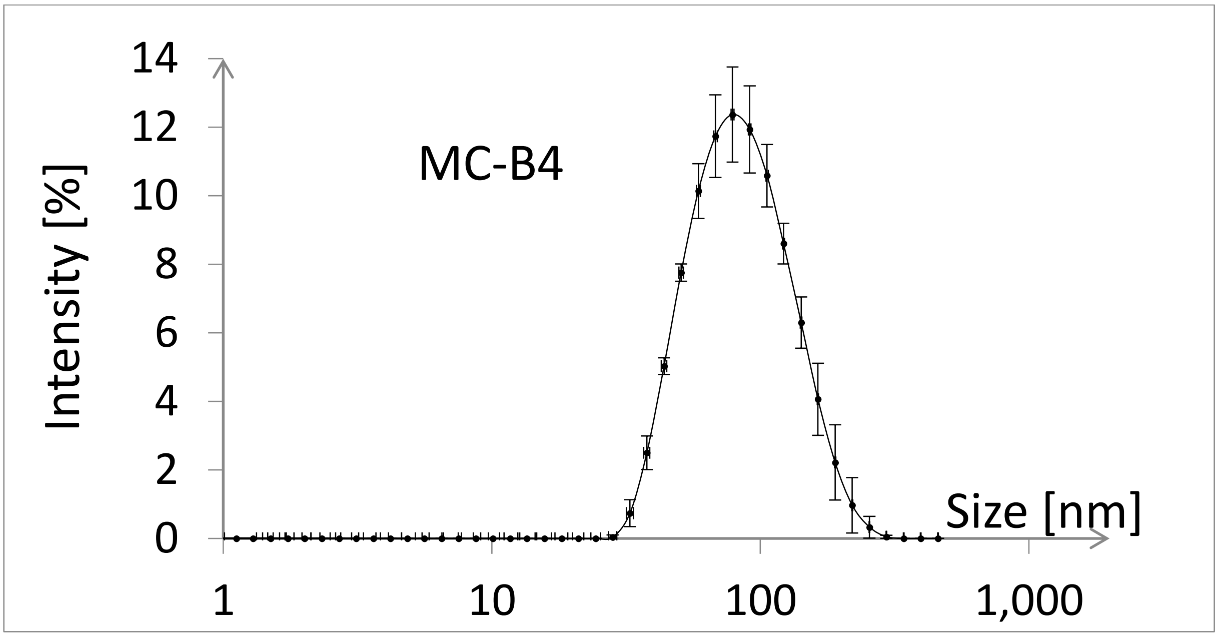

Figure S11: Logarithmic values of the intensity-weighted hydrodynamic size distributions for MC-B4.

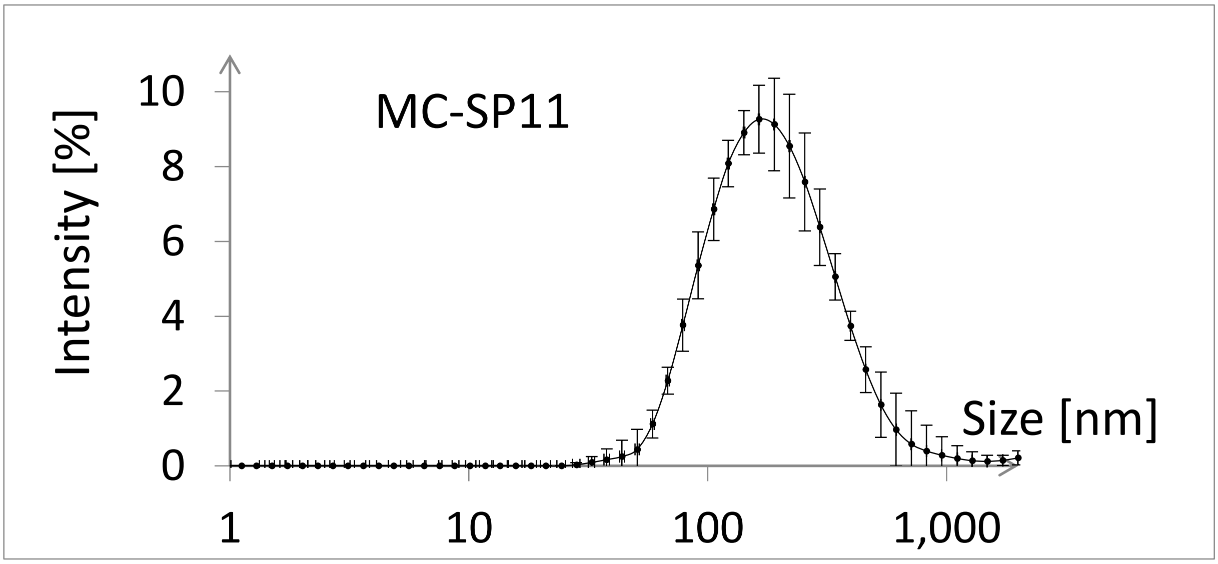

Figure S12: Logarithmic values of the intensity-weighted hydrodynamic size distributions for MC-SP11.
